# Research Progress of Neutrophil-Mediated Drug Delivery Strategies for Inflammation-Related Disease

**DOI:** 10.3390/pharmaceutics15071881

**Published:** 2023-07-04

**Authors:** Yang Zhao, Haigang Zhang, Qixiong Zhang, Hui Tao

**Affiliations:** 1 Department of Pharmaceutics, 96602 Hospital of Chinese People’s Liberation Army, Kunming 650233, China; 2 Department of Pharmacology, College of Pharmacy, Army Medical University, Chongqing 400038, China; 3 Department of Pharmacy, Personalized Drug Therapy Key Laboratory of Sichuan Province, Sichuan Academy of Medical Science & Sichuan Provincial People’s Hospital, Innovation Center of Advanced Pharmaceutical & Artificial Intelligence, School of Medicine, University of Electronic Science and Technology of China, Chengdu 610072, China

**Keywords:** neutrophil, inflammation, targeted drug delivery, biomedical application

## Abstract

As the most abundant white blood cells in humans, neutrophils play a key role in acute and chronic inflammation, suggesting that these cells are a key component of targeted therapies for various inflammation-related diseases. Specific enzyme-responsive or specific ligand-modified polymer nanoparticles are beneficial for improving drug efficacy, reducing toxicity, and enhancing focal site retention. However, there remain significant challenges in biomedical applications of these synthetic polymer nanoparticles, mainly due to their rapid clearance by the reticuloendothelial system. In recent years, biomimetic drug delivery systems such as neutrophils acting directly as drug carriers or neutrophil-membrane-coated nanoparticles have received increasing attention due to the natural advantages of neutrophils. Thus, neutrophil-targeted, neutrophil-assisted, or neutrophil-coated nanoparticles exhibit a prolonged blood circulation time and improved accumulation at the site of inflammation. Despite recent advancements, further clinical research must be performed to evaluate neutrophil-based delivery systems for future biomedical application in the diagnosis and treatment of related inflammatory diseases. In this review, we have summarized new exciting developments and challenges in neutrophil-mediated drug delivery strategies for treating inflammation-related diseases.

## 1. Introduction

Nanomedicine has emerged as an important strategy for targeted drug delivery, which ensures the targeted and precise delivery of drugs to specific cells or tissues [1,2]. Nanoparticles composed of biodegradable polymers, a type of advanced drug delivery system, can significantly improve the pharmacological effects and reduce the potential side effects of drugs [3,4]. The most commonly used polymers in this system include poly(lactic acid), poly(ε-caprolactone), poly(lactide-coglycolide) (PLGA), and poly(alkylcyanoacrylates) [5]. Polymer nanoparticles with a particle size of 10–1000 nm possess some beneficial properties, such as low toxicity, good biocompatibility, and biodegradation. Unfortunately, these synthetic polymer nanoparticles have some limitations, such as short circulation time, low targeting efficiency, and low biological stability. Further, the physical and chemical properties of nanoparticles, including size, shape, charge, and surface chemical properties, affect their cell uptake, tissue accumulation, and adhesion; this facilitates their clearance by the reticuloendothelial system (RES), thereby leading to poor efficiency of targeted delivery [6]. Traditionally, poly(ethylene glycol) (PEG) and polysialic acid have been utilized for the surface functionalization of nanoparticles as the hydration layer which can reduce phagocytosis by RES and significantly extend the blood circulation time [7,8,9]. However, recent studies have suggested that the presence of anti-PEG antibodies enables the rapid clearance of PEG-coated nanoparticles by the liver, thus rendering PEGylation unsuitable for long-term application [10,11]. In addition, bottom-up modification strategies are unfavorable for large-scale production on account of the need for more functional ligands.

**Figure 1:**
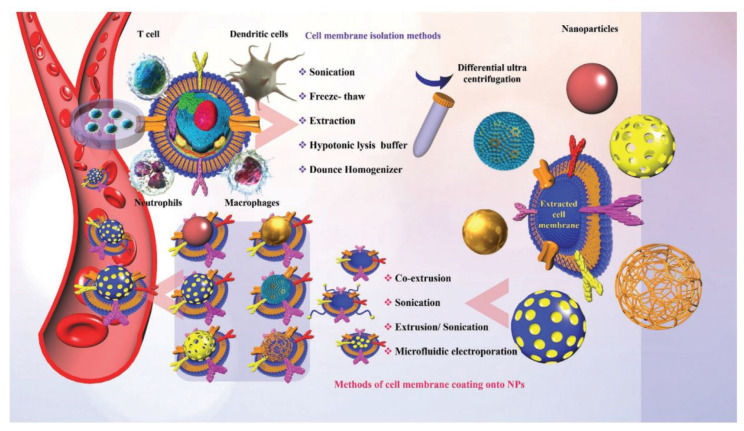
Different source cells, methods of separating cell membranes, and nanoparticles coated with cell membranes. Copyright 2021, Wiley [17].

Surprisingly, biomimetic nanoparticles with the advantages of source cells and synthetic nanoparticles have gained increasing attention [12,13,14]. As one of the most fundamental units in biology, cells have the natural ability to perform complex functions in dynamic environments. Living cells, including erythrocytes, monocytes, macrophages, lymphocytes, neutrophils, and platelets, can be used as naturally biocompatible and degradable drug delivery vehicles that exhibit an inherent capacity to target tissues and cross biological barriers [15]. Furthermore, coating nanoparticles with cell membrane is another biomimetic strategy. Cell-membrane-coated nanoparticles have several advantages, such as extended circulation time and disease-relevant targeting, owing to the inherent properties of the host cell membrane (Figure 1). To date, the membranes of leukocytes, platelets, red blood cells, and cancer cells have been employed for the construction of cell-membrane-coated nanoparticles [16].

**Figure 2:**
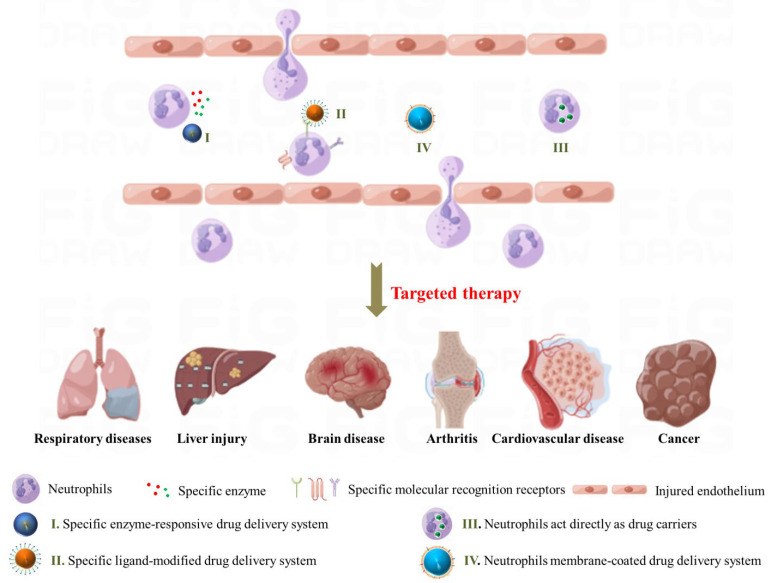
Current strategies on neutrophil-mediated drug delivery for treatment of inflammation-related disease. Image created using Figdraw (www.figdraw.com, accessed on 1 January 2023).

Activated neutrophils play a crucial role in inflammatory diseases. The migration of neutrophils is a highly specific and natural response to inflammation; moreover, neutrophils are often the first immune cells to reach the site of inflammation, making them ideal vectors and targets for drug delivery strategies. As far as we know, the various reviews on neutrophil-mediated targeted delivery systems for nanotherapeutics have been already published [18,19,20,21]. However, so far, there is still a lack of reviews on how to develop neutrophil-targeted, neutrophil-assisted, or neutrophil-coated nanoparticles strategies based on the characteristics of neutrophil-mediated disease. In this review, we summarize the role of neutrophils in inflammation as well as notable findings regarding the use of neutrophil-mediated nanocarriers for targeted drug delivery in different disease models. A search of the literature was performed by examining the PubMed database, and the search terms included “neutrophils”, “target”, and “inflammation”, thus this review is focused on recent progress and promising outcomes of neutrophil-targeted delivery strategies for future biomedical application within the last 10 years from present foreign and domestic research in the English literature, which is important to help researchers understand the opportunities and challenges of neutrophil-based drug delivery systems. For example, we explored specific enzyme-responsive nanoparticles, the surface modification of polymeric nanoparticles with specific ligands (to enhance their ability to target neutrophils), neutrophils as carriers directly, and the latest progress in the biomimetic modification of nanocarriers with neutrophil membranes (summarized in Figure 2 and Table 1). Finally, we discuss the advantages and challenges of neutrophil-mediated drug delivery systems for the diagnosis and treatment of inflammation-related diseases in the future clinical transformation.

## 2. Role of Neutrophils in Diseases

**Figure 3:**
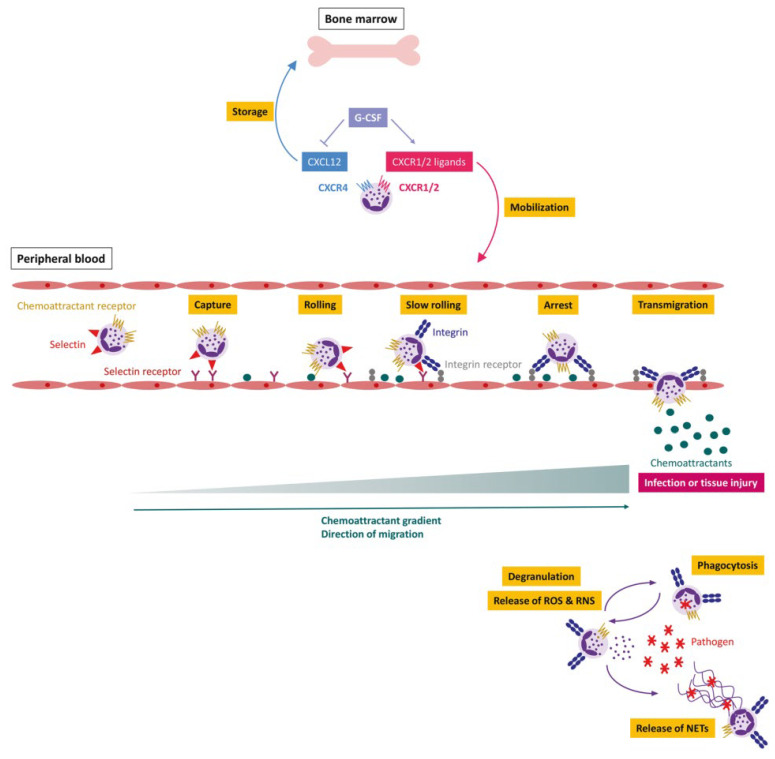
Neutrophil generation, maturation, and egress as well as initiation of inflammation. Copyright 2020, Nature [44].

Inflammation is a defensive process in which the immune system is activated in response to foreign invaders. In general, inflammation is required to protect the host cell from exogenous pathogens and repair damaged tissues. Unfortunately, dysregulated inflammation has been implicated in the pathogenesis of various diseases [33]. In this process, white blood cells infiltrate the tissue and release proinflammatory cytokines, which can cause tissue injury [34]. Neutrophils are the most abundant circulating white blood cells and are considered as the first immune cells to be recruited to an inflamed tissue; as shown in Figure 3, they are generated from hematopoietic stem cells in the bone marrow and granulocyte colony-stimulating factor (G-CSF) drives the granulopoiesis toward neutrophils via the G-CSF receptor [35]. Neutrophil recruitment involves a complex cascade that includes tethering, rolling, adhesion, crawling, and transmigration. This process is specific and highly dependent on the membrane proteins and chemokines [36]. On sensing inflammatory stimuli, they are capable of eliminating the invading pathogens through phagocytosis, degranulation, and the release of neutrophil extracellular traps (NETs) [37]. However, abnormal activation of neutrophils has traditionally been considered to play key roles in acute inflammatory diseases, such as acute peritonitis, acute respiratory distress syndrome, and acute liver injury [22,38]. In recent years, accumulated evidence confirmed that neutrophils are also involved in numerous chronic inflammatory diseases, including chronic respiratory disease, inflammatory bowel disease, rheumatoid arthritis, diabetes, atherosclerosis, and cancer [39,40,41,42,43].

**Figure 4:**
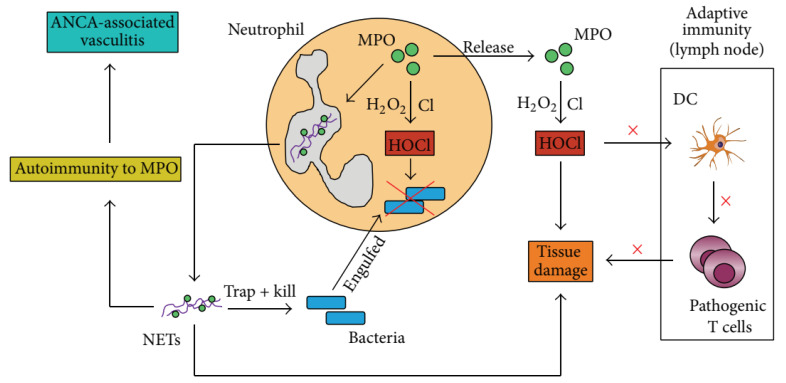
Neutrophils regulate the host immune response. Copyright 2016, Hindawi [53].

In particular, infiltrated neutrophils and their products, including myeloperoxidase (MPO), reactive oxygen species (ROS), and NETs, initiate inflammatory cascades by modulating the innate and adaptive immune responses (Figure 4) [45]. NETs are composed of double-stranded DNA, histones, and granule proteins, including neutrophil elastase (NE), cathepsin G, and MPO. The histones and granule proteins of NETs induce cell apoptosis and promote inflammation. In addition, NETs can activate dendritic cells and T cells to initiate autoimmune responses. Furthermore, NETs activate NLRP3 inflammasome, leading to the maturation and secretion of inflammatory cytokines (IL-1β and IL-18) in macrophages [46]. Consequently, neutrophils play an important role in the initiation of early inflammatory responses and promote the development of inflammatory diseases. For instance, neutrophils and NETs are of crucial importance in acute respiratory distress syndrome. Krishnamoorthy et al. reported that neutrophilic asthma induces neutrophil recruitment and NET formation in the lungs [47]. Soehnlein et al. suggested that neutrophils play a vital role in atherosclerosis [41]. Moreover, a large amount of neutrophils are found in human atherosclerotic plaques, and neutrophil depletion has been shown to reduce early atherosclerosis in mice [48,49]. Further studies have indicated that NETs accelerate the destabilization of atherosclerotic plaques [50]. Additionally, Khandpur et al. demonstrated that neutrophil activation and neutrophil-derived NETs are critically linked to rheumatoid arthritis [51]. Neutrophils also promote both tumor growth and metastasis in cancer [42]. Finally, Albrengues et al. reported that the protumoral effect of neutrophils may be mediated by the release of NETs and suppression of T cell responses [52].

Taken together, these findings demonstrate that neutrophils are vital in various diseases, making them a potential target for therapeutic intervention. There is great potential for the application of the biological characteristics of neutrophils to achieve targeted drug delivery, which should be urgently investigated [54,55].

## 3. Neutrophil-Mediated Drug Delivery Strategies

The current strategies for neutrophil-mediated targeted delivery are summarized based on the following four aspects.

### 3.1. Specific Enzyme-Responsive Drug Delivery System Targeting Neutrophils

**Figure 5:**
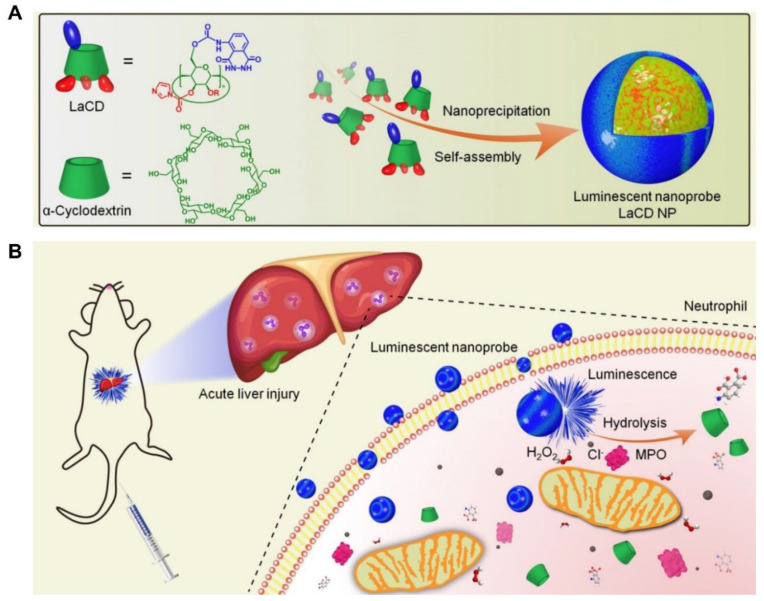
Structure and development of the polymer luminescence nanoprobe (LaCD NP) based on luminol-conjugated α-cyclodextrin (LaCD) (**A**) and specific imaging of infiltrated neutrophils in the injured liver using this nanoprobe (**B**). Copyright 2020, The American Chemical Society [22].

MPO is a heme-containing peroxidase abundantly expressed in neutrophils and mediates a strong oxidative burst through the formation of reactive intermediates. In the presence of H_2_O_2_ and chloride, MPO catalyzes the formation of hypochlorous acid (HClO) and causes significant tissue damage [53]. There is increasing evidence that neutrophil-specific MPO is closely and positively correlated with the risk of progression of many inflammatory diseases [56]. Consequently, an MPO-responsive and biodegradable nanoprobe was designed and constructed, which is utilized for the detection of activated neutrophils during inflammation [38]. For example, luminol is often used to specifically detect MPO activity in neutrophils [57]. It is also used as an effective and biocompatible polymer luminescence nanoprobe (LaCD NP) based on α-cyclodextrin modified with luminol (LaCD), which can be used for in vivo dynamic diagnosis during the development of neutrophil-mediated acute liver injury (Figure 5) [22]. The luminescence of LaCD NP depends on the level of MPO, and the luminescent intensity is positively associated with the number of neutrophils in vitro. Furthermore, in mouse models of acute liver injury, LaCD NP can serve as a luminescent nanoplatform for the noninvasive and real-time detection of neutrophils infiltrated in the liver [22]. This nanoprobe is promising for the diagnosis of other neutrophil-associated diseases and can be utilized as a neutrophil-specific nanocarrier for the targeted delivery of drugs to treat inflammation. MPO-catalyzed HClO is an important ROS in neutrophils, which can promote cell or tissue damage and even cause inflammatory diseases and disorders. Therefore, effective detection of HClO is of great significance for disease research. Hilderbrand et al. developed a MPO-mediated nanoprobe labeled with Alexa Fluor 488 for HClO imaging in a mouse model of myocardial infarction and enabled high-throughput screening of anti-inflammatory molecules [58].

**Figure 6:**
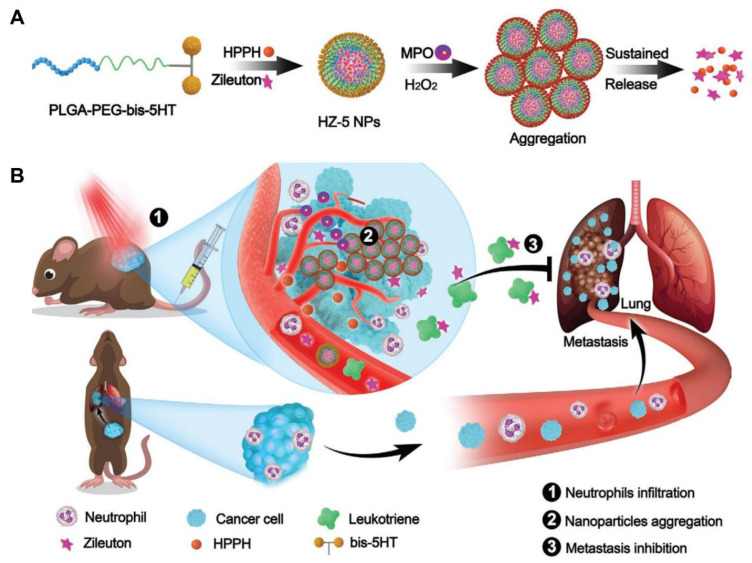
Design and construction of the nanocarrier HZ-5 NPs for MPO-catalyzed aggregation and sustained drug release (**A**) as well as for neutrophil targeting to enhance cancer theranostics (**B**). Copyright 2020, Wiley [23].

Metastasis is the leading cause of death in patients with cancer. This is attributed to the fact that effective treatment is limited once tumors have metastasized from the primary site. However, the current knowledge and development of the cellular and molecular mechanisms involved in tumor metastasis remains incomplete [59]. Recently, numerous studies have elucidated the critical roles of neutrophils in tumor growth and progression. Neutrophils, along with cytokines and chemokines, have a significant impact on the tumor microenvironment, leading to tumor cell proliferation, angiogenesis, and metastasis [60,61]. For example, neutrophils promote the development of lung metastases by releasing proteases and leukotrienes. In addition, tumor-associated neutrophils are capable of suppressing antitumor T cell immunity and assisting tumor cells to escape [62]. Therefore, targeting neutrophils may be an attractive new strategy for metastasis therapy. Notably, Taxiezhung et al. first designed and synthesized neutrophil-targeted nanoparticles via the self-assembly of 5-hydroxytryptamine (5-HT)-conjugated PLGA–PEG–COOH (5HT NP). Subsequently, the photosensitizers 2-(1-hexyloxyethyl)-2-devinyl pyropheophorbide and 5-lipoxygenase inhibitor (zileuton) were loaded onto 5HT NP to obtain the multifunctional nanoparticles HZ-5 NPs (Figure 6) [23]. HZ-5 NPs can specifically target neutrophils through MPO-catalyzed aggregation in tumor tissues, ensuring that drugs loaded onto HZ-5 NPs could be concentrated, retained, and released in a sustained manner at the tumor site, thus achieving effective tumor suppression and resistance against neutrophil-mediated lung metastasis. For an anti-metastatic effect, it is essential to enhance drug accumulation and delivery efficiency at the tumor site. This study developed a new strategy to target neutrophils and enhance the efficacy in tumor treatment, which may provide novel ideas for designing polymer nanoparticles by exploiting the tumor microenvironment.

**Figure 7:**
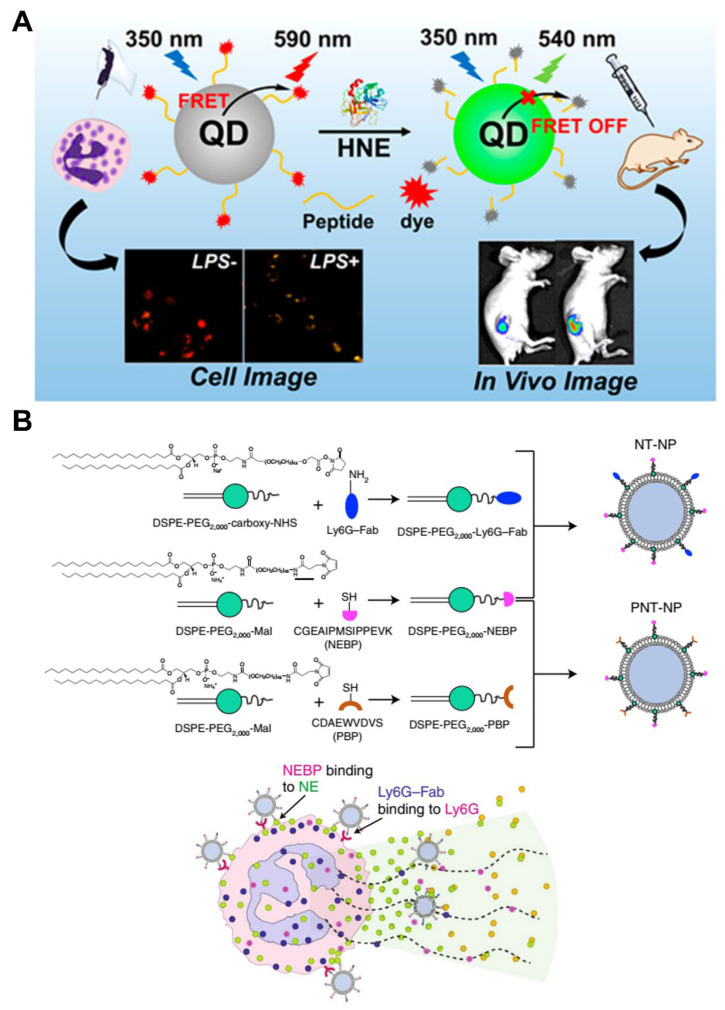
Design and preparation of NE-specific nanoparticles for disease diagnosis (**A**) and treatment (**B**). Copyright 2020, The American Chemical Society [24]; Copyright 2022, Nature [25].

NE is a proteolytic enzyme that is exclusively present on neutrophils, but not on other leucocyte subsets. NE can induce cell proliferation and activate several cytokine and chemokine signaling pathways. The level of NE reportedly correlates with the progression of inflammation [63]. Therefore, NE is considered as a therapeutic target for inflammatory diseases. NE is highly involved in lung inflammatory diseases and lung cancer; accordingly, Liu et al. constructed a nanoprobe based on the fluorescence resonance energy transfer system by incorporating the NE-specific peptide substrate, quantum dots, and organic dyes, providing an applicable tool for in vivo NE detection in mouse models of lung cancer and acute lung injury, thus demonstrating its potential application in the clinical diagnosis of NE-related diseases (Figure 7A) [24]. In addition, Cruz et al. constructed a nanomedicine platform that uniquely utilizes an NE-binding peptide for specific binding to activated neutrophils, which led to a significant reduction in neutrophil activities in vitro and induced the therapeutic effect on murine venous thrombosis in vivo. Thus, it may be a safe and highly efficient approach to neutralize the harmful pathological effects driven by neutrophils, promote healing, and preserve innate immunity or hemostasis (Figure 7B) [25].

Therefore, specific enzyme-responsive nanomaterials play a significant role in neutrophil targeting by taking advantage of the overexpression and increased activity of MPO and NE, which provides a new strategy for treating neutrophil-mediated inflammatory diseases. Nevertheless, there are some challenges associated with the use of these materials. The concentrations of enzymes in the extra- and intracellular milieu should be considered because some materials are extremely sensitive, whereas others require a higher concentration.

### 3.2. Specific Ligand-Modified Drug Delivery System Targeting Neutrophils

In general, by modifying the surface of nanocarriers with specific molecular recognition ligands, the cargo can be delivered to specific cells. This is considered as the most commonly used active targeted delivery strategy. In particular, nanocarriers with controllable and targetable properties can be developed using molecular recognition ligands (antibodies, aptamers, and peptides). Bisso et al. revealed that human serum albumin can modify the surface chemistry of PLGA nanoparticles, thus affecting their uptake by neutrophils [64]. For example, taking advantage of the fact that CD11b is highly expressed in activated neutrophils, nanoparticles modified with an anti-CD11b antibody can be readily ingested by neutrophils in vivo through the recognition of CD11b by anti-CD11b antibody [65].

**Figure 8:**
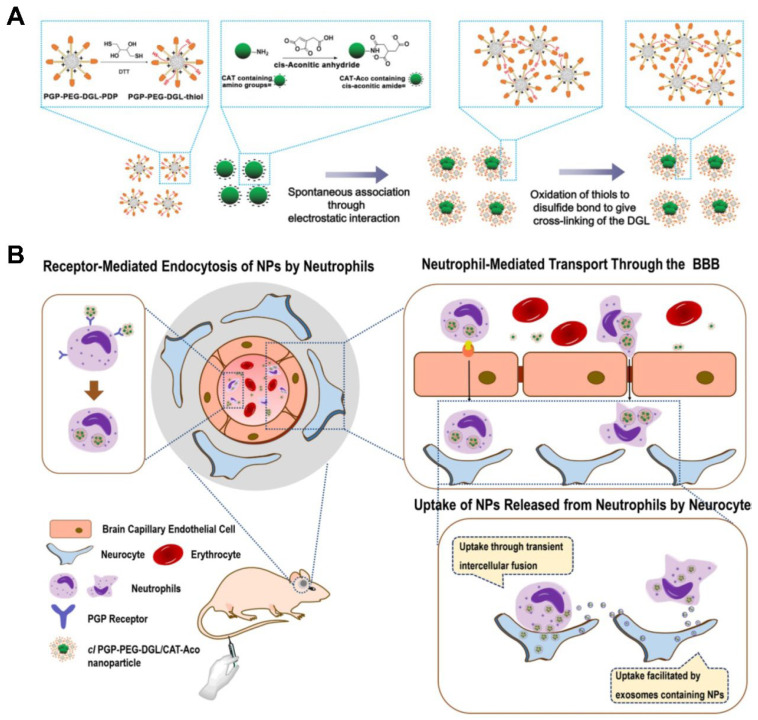
Construction of the *cl* PGP-PEG-DGL/CAT-Aco NP system (**A**) and neutrophil-mediated targeted drug delivery during cerebral ischemia (**B**). Copyright 2017, Ivyspring International [26].

Moreover, Zhang et al. constructed cross-linked dendrigraft poly-l-lysine (DGL) nanoparticles containing *cis*-aconitic anhydride-modified catalase and modified them with the neutrophil-targeting peptide Ac-PGP (*cl* PGP-PEG-DGL/CAT-Aco NPs); this nanoparticle system delivered drugs to cerebral ischemic areas via neutrophil-mediated mechanisms (Figure 8) [26]. In addition, macromolecular drugs have been shown to be promising therapeutics for neurological diseases. However, their instability and poor penetration through the blood–brain barrier (BBB) hinder further application of these agents [66]. Compared with monocytes, an increased number of neutrophils were detected in patients with stroke, which migrate to cerebral ischemic areas in cases of experimental stroke [67,68]. In these studies, neutrophil-targeting NPs exhibited an excellent brain-targeting effect because of the specific phagocytosis induced by endogenous circulating neutrophils, followed by migration across the BBB, which significantly reduced the infarct volume and enhanced the therapeutic outcome of cerebral ischemia [26]. Therefore, this strategy may serve as an effective approach for treating various brain diseases related to neutrophil-mediated inflammation.

**Figure 9:**
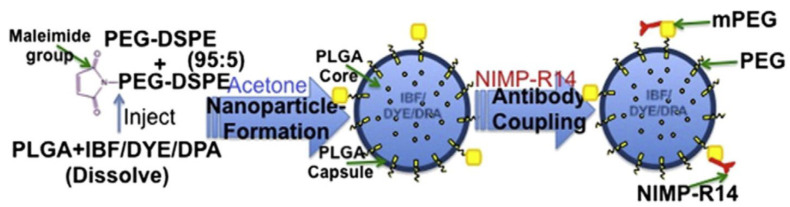
Synthesis of neutrophil-targeted and drug-loaded nanoparticles (PINP^NIMP^) based on a neutrophil-specific antibody. Copyright 2016, Elsevier [27].

Chronic obstructive pulmonary disease (COPD) is a major cause of mortality and morbidity worldwide [69]. In COPD, the levels of neutrophils are elevated, which is associated with the activation of the inflammatory response [70]. Unfortunately, due to the mucus barrier and airway defense, the efficient delivery of therapeutic agents to neutrophils in vivo remains a major challenge. As shown in Figure 9, to avoid rapid clearance and allow access to airway inflammatory cells, Neeraj et al. developed biocompatible and biodegradable neutrophil-targeted polymer nanoparticles consisting of PLGA–PEG nanoparticles conjugated with anti-NIMP-R14 antibodies (PINP^NIMP^) [27]. PINP^NIMP^ can cross the airway barrier and selectively deliver drugs to neutrophils, thus resulting in the alleviation of neutrophilic inflammation in obstructive airway lung diseases. Further preclinical evaluation and standardization are required before employing this strategy for the clinical treatment of pulmonary diseases.

Therefore, surface modification of polymer nanoparticles with specific ligands can effectively target neutrophils to significantly enhance the efficacy of drug delivery and improve therapeutic effects, providing a new avenue for the treatment of various neutrophil-mediated inflammatory diseases. However, these exogenous biofunctionalized polymer nanoparticles cannot mimic the complex intercellular interactions in the human body, resulting in relatively short circulation times and unsatisfactory biodistribution. Thus, new strategies are required to overcome these limitations.

### 3.3. Neutrophils Act Directly as Drug Carriers That Target Diseases

Neutrophils can cross biological barriers and be recruited to inflammatory tissues, making them attractive targeted delivery carriers with excellent movability and biocompatibility [21]. Neutrophils are phagocytes that can uptake different nanoparticles. Nanoparticles can interact with components of the plasma membrane and enter the neutrophils mainly depending on natural endocytosis [71]. Thus, drug carriers based on neutrophils are constructed by incubating nanoparticles with neutrophils, and fluorescence-labeled nanoparticles can be monitored by the live cell imaging system. Consequently, several studies have recently explored the use of neutrophils as vehicles to deliver polymer nanoparticles.

**Figure 10:**
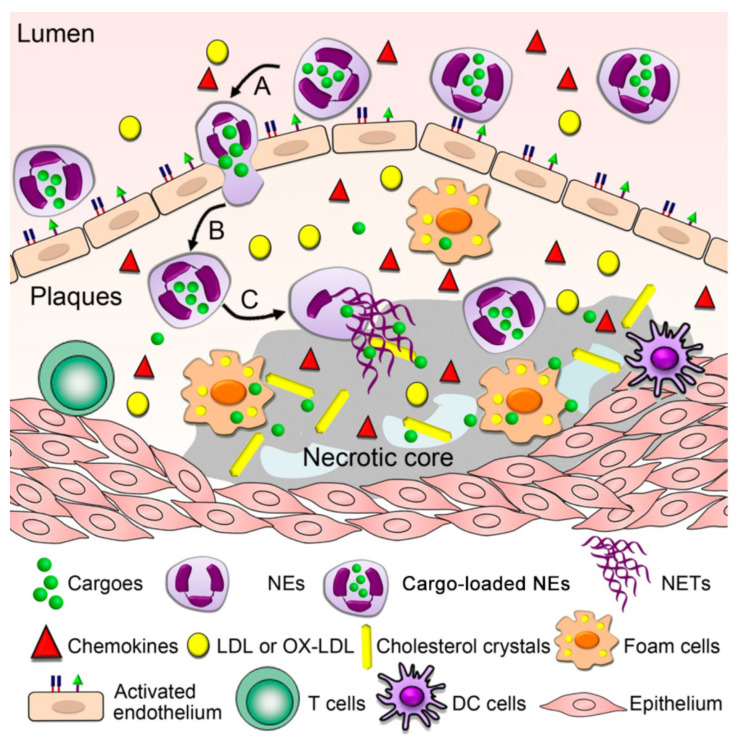
Neutrophils as cellular vehicles loaded with lipid-based polymer nanoparticles for targeting atherosclerotic plaques. Copyright 2019, The American Chemical Society [28].

Atherosclerosis (AS), which is the underlying cause of most cardiovascular diseases, is a chronic inflammatory disease induced by disordered lipid metabolism and imbalanced inflammatory responses and remains the leading cause of morbidity and mortality worldwide [72]. The inflammatory microenvironment of AS plaques consists of various immune cells, such as neutrophils and T lymphocytes; in addition, the important role of neutrophils in the development of AS has been well documented [41,73]. Many studies have suggested that adhesion molecules, including E-selectin, P-selectin, ICAM-1, and CXCL1/KC, which are overexpressed in the activated vascular endothelium, can recruit neutrophils to the atherosclerotic lesions [74,75]. Therefore, as shown in Figure 10, neutrophils may be used as cellular carriers to target AS plaques, which can effectively avoid phagocytosis in the liver and spleen and further overcome the challenges of the traditional nanomedicine therapy for AS.

**Figure 11:**
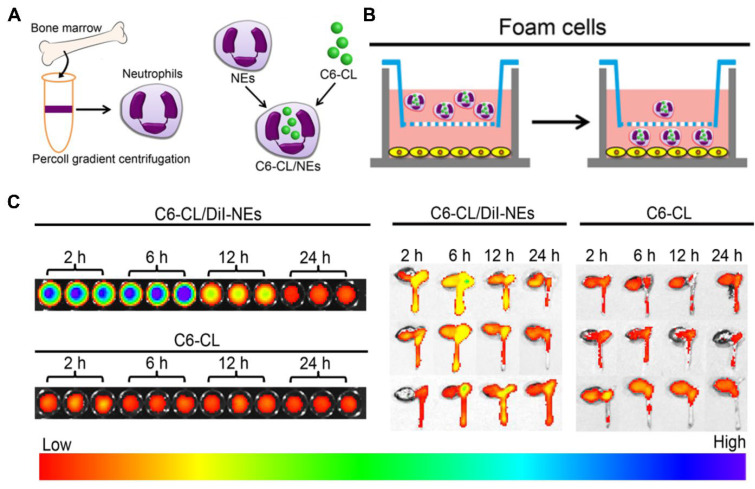
Procedure used for neutrophil isolation and the construction of cellular vehicles based on neutrophils (**A**), in vitro targeted delivery of cellular vehicles based on neutrophils (**B**), and in vivo atherosclerotic plaque-targeting effect of cellular vehicles based on neutrophils in ApoE^−/−^ mice with atherosclerotic lesions (**C**). Copyright 2019, The American Chemical Society [28].

In this context, Xue et al. exploited neutrophils as cellular vehicles to load a cationic lipid polymer of 1,5-dioctadecyl-*N*-histidyl-l-glutamate (HG2C_18_)-based nanoparticles, thereby targeting atherosclerotic sites (Figure 10) [28]. Neutrophils were first collected from the bone marrow and then utilized to load fluorescent-labeled nanoparticles as cargos, which can specifically target foam cells in vitro (Figure 11A,B). In an animal model of AS, the use of neutrophils as cellular carriers allowed the effective and specific delivery of nanoparticles to the site of atherosclerotic plaques (Figure 11C). In this strategy, the property of natural chemotaxis of neutrophils in AS plaques was exploited, ensuring that the nanoparticles loaded in the neutrophils can be recruited to atherosclerotic lesion sites. In addition, this strategy has been applied in targeted therapy for cancer [76]. Accordingly, we believe that cellular vehicles based on neutrophils are promising and excellent carriers that can be used to load agents or nanoparticles for treating other neutrophil-mediated diseases.

**Figure 12:**
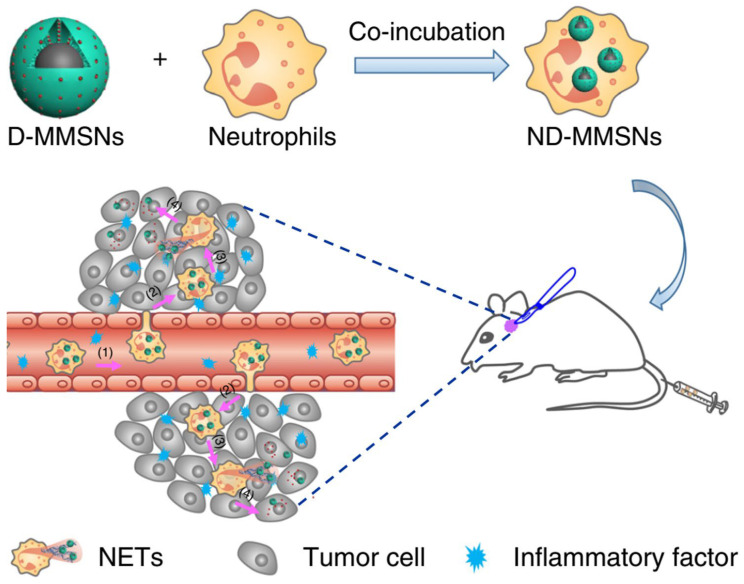
Schematic illustration of the fabrication of ND-MMSNs and their targeted therapy for inflamed glioma. Copyright 2018, Nature [29].

Gliomas, which are the most common primary tumors of the central nervous system, have a poor prognosis and high mortality. Nevertheless, because of the highly aggressive and infiltrative nature of glioma cells, the therapeutic effect of surgical resection is limited. In addition, the effect of chemotherapy is hindered by the limited penetration of drugs across the blood–brain barrier and blood–tumor barrier [77,78,79]. Even though the blood–brain barrier can be overcome using a polymer nanoparticle-based drug delivery system with an active targeting function, its therapeutic effect remains unsatisfactory owing to a poor blood circulation lifespan and insufficient intertumoral drug accumulation [80]. Gratifyingly, immune-cell-based drug delivery vehicles can assist drugs in crossing the vascular barrier and migrating to the tumor sites, thereby reducing immune clearance and extending the biological half-life of the drugs [81,82]. Neutrophils play a critical role during cancer progression and metastasis, and they possess the natural ability to traverse the blood–brain barrier and infiltrate tumor tissues; thus, they can serve as “living” vehicles to carry cargos to the brain. Recently, Wu et al. reported a strategy for the targeted delivery of doxorubicin-loaded magnetic mesoporous silica nanoparticles using neutrophils as cellular carriers (ND-MMSNs) for glioma theranostics (Figure 12) [29].

**Figure 13:**
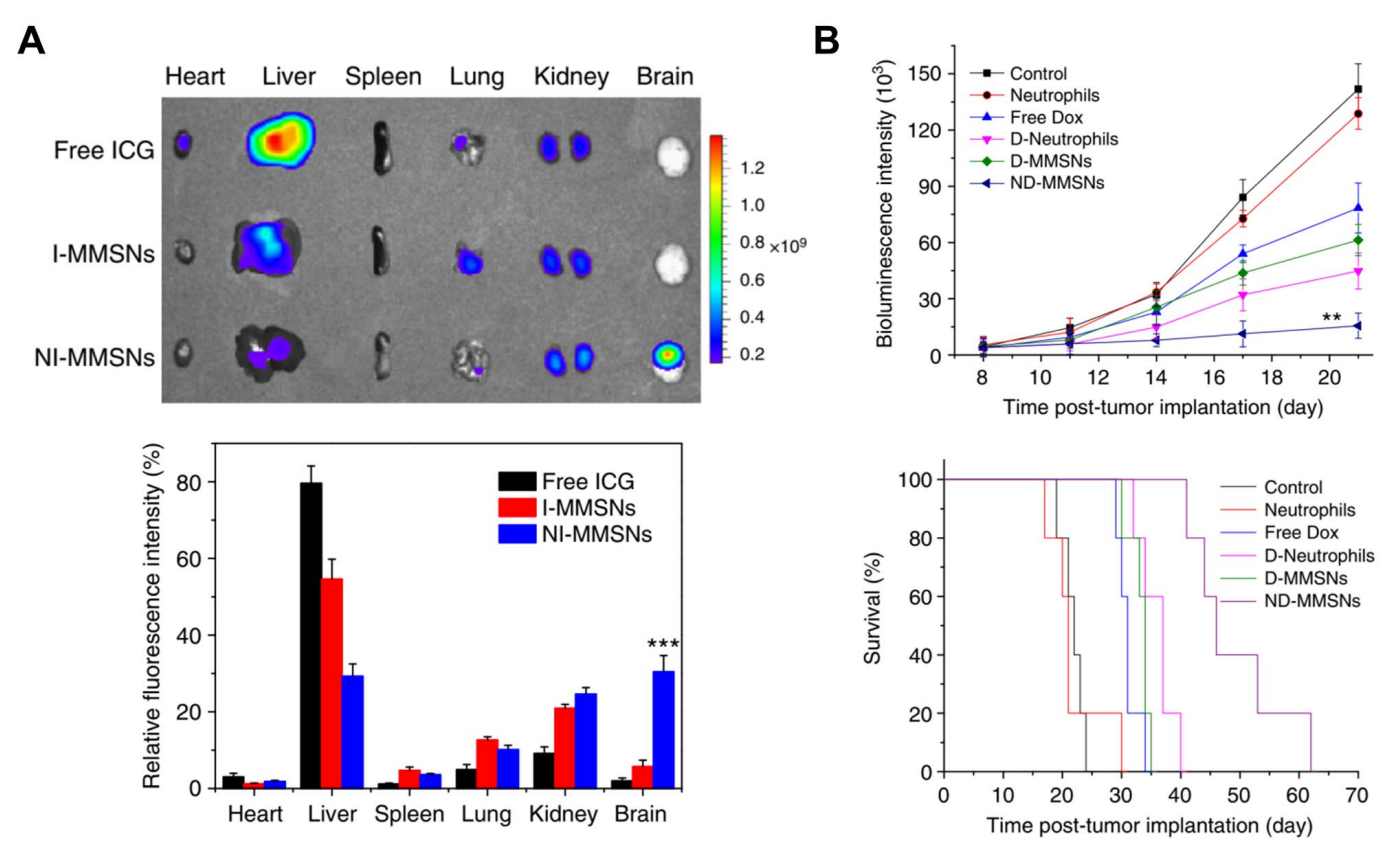
In vivo biodistribution of cellular carriers in major organs excised from mice intravenously injected with free ICG, I-MMSNs, and NI-MMSNs at 4 h postinjection (**A**), and in vivo therapeutic effect of various treatments on C6 glioma-bearing mice in each group (**B**). ** *p* < 0.01 *** *p* < 0.001. Copyright 2018, Nature [29].

MMSNs were synthesized by encapsulating magnetic Fe_3_O_4_ nanoparticles in mesoporous silica using polymer cetyltrimethylammonium bromide as the surfactant. Similarly, MMSNs loaded with the near-infrared dye indocyanine green (ICG) were prepared (I-MMSNs) and then co-incubated with neutrophils to form NI-MMSNs. In the incomplete resection glioma model, NI-MMSNs exhibited a significantly higher targeted accumulation in the brain than free ICG and I-MMSNs after injection (Figure 13A). Consequently, ND-MMSNs exhibited the best inhibitory effect on tumor regeneration and significantly prolonged the postoperative survival time in C6 glioma-bearing mice, indicating that doxorubicin was increasingly accumulated at the glioma sites via neutrophils (Figure 13B). Therefore, their research offers a new approach for targeted cancer therapy by combining the advantages of neutrophils and polymer nanoparticles.

Unlike the traditional nanoparticles that target accumulation at the inflammatory site based on responsive targeting, known as enzyme stimulation, or active targeting via ligand–receptor interactions, cellular vehicles based on neutrophils can overcome the challenges of nanomedicines. Neutrophils in the blood can effectively avoid phagocytosis in the liver and spleen. Thus, neutrophils as drug carriers provide new strategies for treating neutrophil-mediated inflammatory diseases. Conversely, the recruitment of neutrophils is a natural process, which could result in their enrichment at the inflammatory site. Further research needs to be performed to evaluate the application of neutrophil hitchhiking in inflammatory diseases.

### 3.4. Neutrophil-Membrane-Coated Drug Delivery System for Disease Targeting

The use of natural living neutrophils as drug delivery carriers is limited by their poor viability after isolation. The cell membrane coating technology was first reported in 2011, in which the whole cell membrane was modified onto the surface of nanoparticles. The resulting membrane-coated nanoparticles are capable of mimicking the source cells, thereby extending their circulation time, alleviating immune recognition, and transporting therapeutics to the inflamed focus [83]. Currently, neutrophil-membrane-coated nanoparticles are gaining considerable interest for the treatment of diverse neutrophil-associated acute and chronic inflammatory diseases.

**Figure 14:**
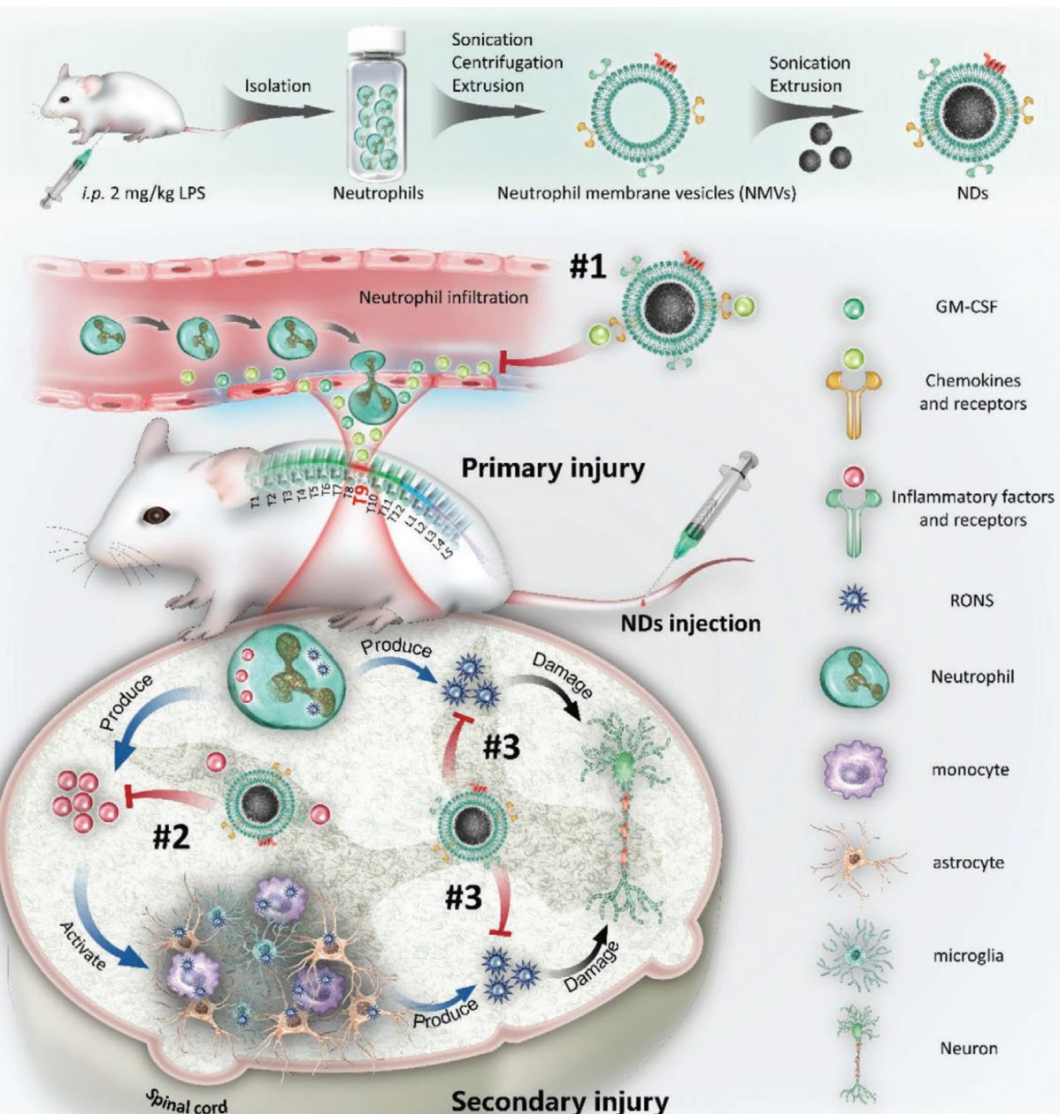
Schematic illustration showing the preparation of NDs and their application in targeted therapy for the secondary injury of SCI. #1 NDs reduce local neutrophil infiltration by adsorbing chemokines. #2 NDs neutralize inflammatory factors to alleviate the activation of cells and secondary injury. #3 The polydopamine (PDA) NPs coating NDs relieve oxidative stress in the injured spinal cord after SCI. Copyright 2021, Wiley [30].

Traumatic spinal cord injury (SCI) represents a significant cause of neuron death, spinal cord damage, and even permanent paralysis. During SCI, neutrophils infiltrate the injured spinal cord and release various inflammatory mediators, which further induce a secondary injury [84,85]. However, the previously reported single receptor targeted therapy for SCI remains unsatisfactory for inhibiting the proinflammatory function of neutrophils [86,87,88]. Bi et al. constructed a type of neutrophil decoy (ND) by coating polydopamine nanoparticles with neutrophil membranes, which can inhibit neutrophil activity and significantly promote neural regeneration and functional recovery after SCI (Figure 14) [30].

**Figure 15:**
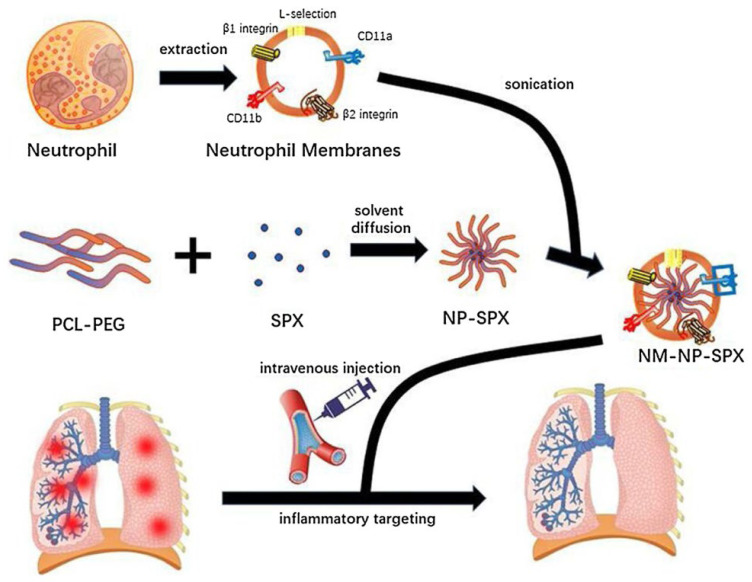
Schematic diagram of NM-NP-SPX and the targeted therapy for pneumonia. Copyright 2019, Elsevier [31].

Multiple receptors derived from source neutrophils on the surface of NDs can help them migrate to the injured spinal cord and neutralize neutrophil-associated chemokines and cytokines. Moreover, Feng et al. constructed a neutrophil-like cell-membrane-coated mesoporous Prussian blue nanozyme using potassium ferricyanide and polyvinylpyrrolidone, which can accumulate in the injured brain, to treat ischemic stroke based on the specific interaction between neutrophils and inflamed brain microvascular endothelial cells after stroke [89]. To improve the treatment effect on pneumonia, Wang et al. designed neutrophil-membrane-coated sparfloxacin-loaded polymer nanoparticles (denoted as NM-NP-SPX) that can imitate activated neutrophils and precisely accumulate in the inflammatory lungs (Figure 15) [31]. Compared with traditional polymer nanoparticles, NM-NP-SPX prolonged the circulation time and reduced cytotoxicity. Furthermore, NM-NP-SPX could efficiently alleviate lung inflammation in the mouse pneumonia model. Taken together, their results lay the foundation for further effective promotion of functional recovery in patients with acute inflammatory diseases using neutrophil-based biomimetic nanoplatforms.

**Figure 16:**
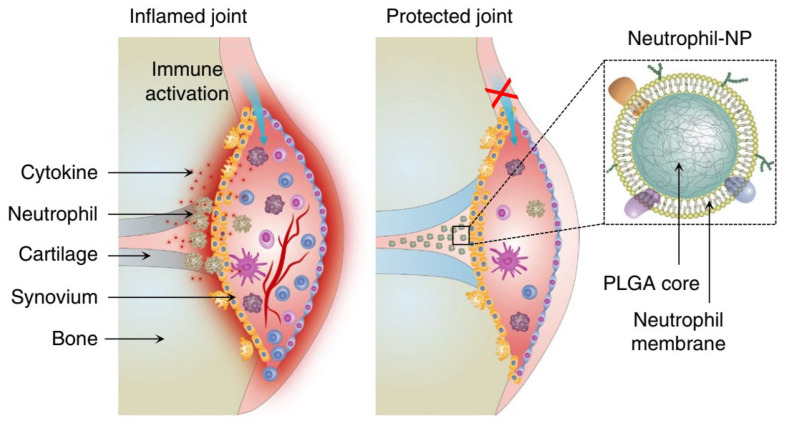
Schematic representation of neutrophil-like nanoparticles for the targeted treatment of joint destruction in patients with rheumatoid arthritis. Copyright 2018, Nature [32].

Rheumatoid arthritis, a common chronic disease characterized by systemic inflammation, is a major cause of disability. The current treatments are primarily directed at inflammatory responses using biologics that interfere with the production and functions of various cytokines [90,91,92]. However, there remains a pressing need for better therapies for rheumatoid arthritis, considering its adverse effects and safety concerns. Several studies have suggested that neutrophil infiltration and activation in the joints of patients with rheumatoid arthritis contribute directly to bone destruction [93]. Therefore, Zhang et al. constructed neutrophil-like nanoparticles by modifying the surface of PLGA nanoparticles with neutrophil membranes, which can deplete immunoregulatory molecules to dampen the inflammation, thus providing a novel anti-inflammatory strategy for managing rheumatoid arthritis (Figure 16) [32]. Similarly, Kang et al. developed a neutrophil-mimicking drug delivery system loaded with carfilzomib that enabled the accumulation of carfilzomib at lesion sites and further inhibited tumor metastasis [94]. The biomimetic drug delivery system will provide an advanced strategy to alleviate inflammation in patients with chronic inflammatory diseases.

In the abovementioned studies, neutrophil membranes were derived from human or mouse whole blood, which hinders the large-scale production and clinical application of the corresponding nanoparticles in the future. Encouragingly, significant advances have been reported in the generation of immunocompatible cells, which overcome the limitation of the large-scale production of neutrophils and enable clinical research [95,96]. Therefore, we believe that the biomimetic strategy using neutrophil membrane-coating technology can improve the in vivo specific distribution and targeted therapeutic effect of polymer nanoparticles, which may be applicable to the treatment of various inflammatory diseases.

## 4. Conclusions and Perspectives

We summarized the recent progress in the field of neutrophil-based polymer nanocarriers and revealed that compared with traditional treatments, novel biomimetic technologies can dramatically enhance the therapeutic efficacy in the context of various inflammatory disorders and diseases by taking advantage of neutrophils or neutrophil membranes as carriers. A comparison of different neutrophil-mediated nanodrug delivery systems is shown in Table 2.

Neutrophils are key effector cells in the inflammatory response and accumulate at sites of inflammation, making them represent a new therapeutic approach for the treatment of inflammatory disorders. Polymer nanoparticles serve as excellent vehicles for the site-specific delivery of drugs to the target cells or tissues by modifying the polymer through physical or chemical means. However, few neutrophil-targeting strategies are available at present. The rationally designed nanocarriers with suitable surface ligands conjugated to the molecules expressed on neutrophils to increase their internalization should be explored in future studies. In addition, versatile and reliable visualization of neutrophils under different inflammatory conditions is necessary for the precise treatment of diseases. Multifunctional theranostic polymer nanoparticles should be designed for site-specific delivery of drugs and contrast agents for multimodality molecular imaging of the processes involved in neutrophil-based combination therapy. Unfortunately, synthetic polymer nanoparticles are inherently foreign and fail to imitate the complex functions of biological systems. To overcome these limitations, neutrophil-based biomimetic nanoparticles represent more favorable drug delivery systems with excellent targeting capabilities, enhanced stability, and lower toxicity. Regarding the design and application of neutrophil-based biomimetic nanoparticles, several caveats should be addressed to ensure effective treatments. First, synthetic polymer nanoparticles can be readily ingested by neutrophils in vitro. The size, shape, and charge of polymer nanoparticles have a strong effect on the capacity of neutrophils to ingest them. Secondly, smaller nanoparticles can reduce their impact on the migration ability, survival ability, and biological function of neutrophils extracted in vitro. This is crucial for effectively delivering cargoes to the target site in the body. Third, it should be ensured that synthetic polymer nanoparticles are not degraded significantly until they reach the target sites, implying that the biomimetic nanoplatform should be stored for a prolonged time with excellent stability. Moreover, ideal manufacturing processes are required to ensure that the final product is free of chemical and biological contaminants and to enable large-scale production.

The non-specific distribution of small-molecule drugs causes significant off-target toxicity at therapeutic doses and they are easily cleared. Compared to small molecules, nanoparticles can improve the stability and solubility of drugs and display unique pharmacokinetics (PK) and biodistribution (BD), which can increase blood circulation and safety [97]. Biodegradable polymers and some natural polymers are widely used to reduce toxicity and increase biocompatibility in the development of nanoparticles. Targeting nanoparticles may allow an active accumulation at desired sites and enhance local drug concentration, thus avoiding systemic side effects and increasing retention time by conjugation with specific ligands or stimuli-responsive linkers [98]. Immune cells exhibit high biocompatibility, longer circulation times, inherent biodegradability, and a natural ability to target cells/tissues due to their unique structure and surface function [15]. Therefore, coating the neutrophil membrane on the surface of nanoparticles or neutrophils as carriers enables biomimetic nanoparticle to have both the physicochemical properties of the nanoparticle and the biological functions of the neutrophils, which shows prolonged blood circulation and low toxicity.

Although neutrophils-mediated drug delivery strategies have shown promise in preclinical models for improving the treatment effect of various diseases as mentioned above, whether the neutrophil-based delivery system is safe and effective in clinical application remains controversial. Furthermore, large-scale production and reproducibility are obstacles to clinical transformation. Consequently, further clinical research must be performed to evaluate the application of neutrophil-based delivery systems in various inflammatory diseases. Despite current challenges, neutrophil-mediated polymer nanoparticles emerge as promising therapeutics for diverse acute and chronic inflammatory diseases. Advances in polymer nanomaterials and biomimetic nanotechnology may facilitate the construction of neutrophil-based multifunctional nanoplatforms for biomedical research and applications in the future. Taken together, neutrophil-based drug delivery systems will have a significant impact on nanomedicine.

## Figures and Tables

**Table 1:** Summary of the neutrophil-mediated drug delivery strategies for treatment of inflammation-related disease.

Targeting Strategies	Mechanisms	Applications	Ref.
Specific enzyme-responsive drug delivery system	MPO and NE are specific enzymes abundantly expressed in neutrophils and mediate inflammation	Acute liver injury, lung metastasis, venous thrombosis	[22,23,24,25]
Specific ligand-modified drug delivery system	Specific molecular recognition ligands (antibodies, aptamers, and peptides) can be bound to neutrophils	Cerebral ischemia, COPD	[26,27]
Neutrophils act directly as drug carriers	Neutrophils can cross biological barriers and be recruited to inflammatory tissues	Atherosclerosis, gliomas	[28,29]
Neutrophil-membrane-coated drug delivery system	Neutrophil membranes are capable of mimicking the source cells and transporting to the inflamed focus	Traumatic spinal cord injury, pneumonia, rheumatoid arthritis	[30,31,32]

**Table 2:** A comparison of different neutrophil-mediated nanodrug delivery systems.

Nanodrug	Delivery Strategies	Targeting	Challenges	Ref.
HZ-5 NPs	Specific enzyme-responsive drug delivery system	Specifically target neutrophils through MPO-catalyzed aggregation	The concentrations of enzymes should be considered	[23]
PINP^NIMP^	Specific ligand-modified drug delivery system	Anti-NIMP-R14 antibodies can effectively target neutrophils	Cannot mimic the complex intercellular interactions	[27]
ND-MMSNs	Neutrophils act directly as drug carriers	Neutrophils can cross biological barriers and be recruited to inflammatory tissues	Living neutrophils as drug delivery carriers are limited by their poor viability after isolation	[29]
NM-NP-SPX	Neutrophil-membrane-coated drug delivery system	The resulting membrane-coated nanoparticles are capable of mimicking the source cells	Complicated, careful manufacturing processes and should be stored for a prolonged time with excellent stability	[31]

## Data Availability

Not applicable.
